# The key hypoxia regulated gene *CAIX* is upregulated in basal-like breast tumours and is associated with resistance to chemotherapy

**DOI:** 10.1038/sj.bjc.6604844

**Published:** 2009-01-22

**Authors:** E Y Tan, M Yan, L Campo, C Han, E Takano, H Turley, I Candiloro, F Pezzella, K C Gatter, E K A Millar, S A O'Toole, C M McNeil, P Crea, D Segara, R L Sutherland, A L Harris, S B Fox

**Affiliations:** 1 Nuffield Department of Clinical Laboratory Sciences, John Radcliffe Hospital, Oxford OX3 9DU, UK; 2 Pathology, Peter MacCallum Cancer Centre, St Andrews Place, East Melbourne 3002, Australia; 3 Pathology, University of Melbourne, Melbourne, Victoria, Australia; 4 Cancer Research UK Molecular Oncology Laboratory, Weatherall Institute of Molecular Medicine, John Radcliffe Hospital, Oxford OX3 9DU, UK; 5 Cancer Research Program, Garvan Institute of Medical Research, 384 Victoria Street, Darlinghurst, NSW 2010, Australia; 6 Department of Anatomical Pathology, South Eastern Area Laboratory Service, St George Hospital, Kogarah, NSW 2217, Australia; 7 Department of Pathology (Sydpath), St Vincent's Hospital, Darlinghurst, NSW 2010, Australia; 8 Department of Medical Oncology, Westmead Hospital, Sydney, NSW, Australia; 9 Department of Surgical Oncology, St Vincent's Clinic, Darlinghurst, NSW, Australia

**Keywords:** breast, hypoxia, carbonic anhydrase, predictive, basal

## Abstract

Basal-like tumours account for 15% of invasive breast carcinomas and are associated with a poorer prognosis and resistance to therapy. We hypothesised that this aggressive phenotype is because of an intrinsically elevated hypoxic response. Microarrayed tumours from 188 patients were stained for hypoxia-inducible factor (HIF)-1*α*, prolyl hydroxylase (PHD)1, PHD2, PHD3 and factor inhibiting HIF (FIH)-1, and carbonic anhydrase (CA) IX stained in 456 breast tumours. Tumour subtypes were correlated with standard clincopathological parameters as well as hypoxic markers. Out of 456 tumours 62 (14%) tumours were basal-like. These tumours were positively correlated with high tumour grade (*P*<0.001) and were associated with a significantly worse disease-free survival compared with luminal tumours (*P*<0.001). Fifty percent of basal-like tumours expressed HIF-1*α*, and more than half expressed at least one of the PHD enzymes and FIH-1. Basal-like tumours were nine times more likely to be associated with CAIX expression (*P*<0.001) in a multivariate analysis. Carbonic anhydrase IX expression was positively correlated with tumour size (*P*=0.005), tumour grade (*P*<0.001) and oestrogen receptor (ER) negativity (*P*<0.001). Patients with any CAIX-positive breast tumour phenotype and in the basal tumour group had a significantly worse prognosis than CAIX-negative tumours when treated with chemotherapy (*P*<0.001 and *P*=0.03, respectively). The association between basal phenotype and CAIX suggests that the more aggressive behaviour of these tumours is partly due to an enhanced hypoxic response. Further, the association with chemoresistance in CAIX-positive breast tumours and basal-like tumours in particular raises the possibility that targeted therapy against HIF pathway or downstream genes such as CAs may be an approach to investigate for these patients.

Current prognostic and predictive markers of invasive breast cancer rely largely on histological indicators such as tumour size, grade, nodal status and receptor status, which define broad patient groups for different treatment regimens. However, it is recognised that there are many patients within such groups who are overtreated or undertreated with conventional therapies, strongly suggesting that current stratification does not fully account for the molecular heterogeneity and diverse biological behaviour of breast cancer. Recently cDNA microarray studies have classified breast cancers broadly into five subtypes, based on their different expression profiles: normal breast-like, luminal A (oestrogen receptor (ER) positive), luminal B (ER positive and proliferative), HER2 overexpressing and basal (Sorlie *et al*, 2001, 2003), the latter having a particularly poor clinical outcome (Sorlie *et al*, 2003; Abd El-Rehim *et al*, 2004; Nielsen *et al*, 2004).

Basal-like breast cancers derive their name from their characteristic expression of basal and myoepithelial markers such as cytokeratins (CK) 5, 14 and 17 (Jarasch *et al*, 1988; Wetzels *et al*, 1991). These tumours account for up to 15% of all invasive breast cancers (van de Rijn *et al*, 2002; Abd El-Rehim *et al*, 2004; Nielsen *et al*, 2004; Matos *et al*, 2005). Basal-like tumours are more frequently observed in patients with BRCA1-related cancers (Foulkes *et al*, 2004). In contrast to the normal breast-like and luminal subtypes, basal-like breast cancers have a particularly aggressive phenotype, being more likely to be high-grade, ER negative (Malzahn *et al*, 1998; Korsching *et al*, 2002; Nielsen *et al*, 2004), and are associated with a higher proliferative index (Korsching *et al*, 2002; Matos *et al*, 2005). Expression of basal CKs is also associated with loco-regional recurrence, distant metastasis and a poor overall survival in node-positive and node-negative patients (Malzahn *et al*, 1998; van de Rijn *et al*, 2002). In addition, basal-like tumours differ from normal breast-like and luminal tumours in their response to chemotherapy. Although basal-like tumours do not respond well to anthracycline-based therapy (Banerjee *et al*, 2006), they show an impressive pathological response to paclitaxel followed by a combination of 5-fluorouracil, doxorubicin and cyclophosphamide (Rouzier *et al*, 2005). They also have a BRCA1 phenotype and therefore are sensitive to platinum-based therapies (Kennedy *et al*, 2004).

In addition to being mostly high-grade tumours, a so-called triple negative phenotype (ER, progesterone receptor (PR) and HER2 negative), basal-type breast cancers may also have a characteristic central acellular or necrotic zone (Tsuda *et al*, 1999; Fulford *et al*, 2006). Tumour necrosis is a consequence of hypoxia. Hypoxia is a predictor of poor disease-free and overall survival in breast cancer (Fisher *et al*, 1993; Hockel and Vaupel, 2001), and is associated with resistance to radiation and certain chemotherapeutic regimes (Teicher *et al*, 1990). As the hypoxia-inducible factor (HIF) pathway has been shown to be pivotal in the hypoxic tumour response and to mediate many of the above adverse effects, and in view of the link between basal-like breast cancer, hypoxia and an aggressive phenotype, we have examined the HIF pathway, including the expression of HIF-1*α*, its regulatory enzymes the prolyl hydroxylase domain enzymes PHD-1, PHD-2, PHD-3, factor inhibiting HIF-1 (FIH-1) and carbonic anhydrase (CA) IX, a downstream target of HIF-1*α* in a series of breast cancers, and compared the expression of each marker by correlating it with various standard clinicopathological parameters.

## Materials and methods

### Patient characteristics

Tumour tissue microarray cores (1 mm cores) were collected from 621 invasive breast carcinomas collected from patients who had undergone surgery at the John Radcliffe Hospital, Oxford, UK and from the Garvan Institute, Sydney, Australia. This study has Ethical Committee approvals (number JR C02.216 and HREC SVH H94/080, HREC SVH 06336 H00036). All patients had operable breast carcinomas and were not diagnosed with metastatic disease at the time of presentation. Information regarding patient characteristics, including age, tumour size, grade, histology, nodal status, ER and HER2 status, were collected from the clinical and pathological records. The median age of patients included in this study was 55 years (range 24–87 years). Eighty-eight percent of tumours were invasive ductal of no specific type, 3% were invasive lobular carcinoma and 3% were of other histological classifications (data was unavailable for the remaining 6%). Median tumour size was 20 mm and the median tumour grade was two. Forty-four percent of patients had nodal disease. Sixty-eight percent of tumours were ER positive and 16% were HER2 positive. Patients less than 50 years of age with node-positive, ER-negative tumours or tumours larger than 3 cm received adjuvant chemotherapy (cyclophosphamide, methotrexate and 5-fluorouracil (CMF) or adriamycin and cyclophosphamide (AC). Patients with hormone-responsive tumours who were more than 50 years of age received 5 years of endocrine therapy. Patients were followed up for a median period of 131.9 months. During this time, 137 patients developed recurrence (30.0%) and 99 deaths (21.7%) were considered as breast-cancer related.

### Immunohistochemistry

Formalin-fixed paraffin-embedded tissue sections were stained for the various hypoxic markers (Supplementary Table 1). Substitution of the primary antibody with phosphate-buffered saline served as a negative control. Transfected COS-1 cells expressing PHD1, PHD2, PHD3, FIH-1 and HIF-1*α* were used as positive controls. In each run a tumour is known to be positive for CAIX was included.

Scoring was carried out by two observers simultaneously. ER, HER2, EGFR and CK5/6 staining was used to classify tumours into four subgroups as per Neilson *et al*. basal group (ER negative, HER2 negative, CK5/6 and/or EGFR positive), luminal group (ER positive, HER2 negative), HER2 group (HER2 positive) and a negative group (ER negative, HER2 negative, CK5/6 negative and EGFR negative) (Nielsen *et al*, 2004). Tumours with any nuclear staining for HIF-1*α* were considered positive in the analysis. The levels of staining for PHD1, PHD2 and PHD3 were scored with respect to the percentage of cells and the intensity of staining in the cytoplasm. The scoring system for intensity was: 0=no staining, 1=weak staining, 2=moderate staining, 3=strong staining; and the scoring system for percentage was: 0=no cells staining positive, 1=⩽10% cells staining positive, 2=11–50% positive cells, 3=51–80% positive cells, 4=⩾80% positive cells. Tumours with a cytoplasmic intensity score of ⩾2 and a percentage score of ⩾1 were considered positive for PHD1, PHD2 and PHD3 in the analysis, in accordance with their site of action. Only tumours showing a strong membranous staining in ⩾10% or more cells were considered positive for CAIX (Tan *et al*, 2007). Tumours with a nuclear intensity score of ⩾2 and a percentage score of ⩾1 were considered positive for FIH-1 in the analysis (Tan *et al*, 2007). Owing to loss of tumour cores, only 456 out of 621 patients with full phenotype of ER, HER2, EGFR and CK5/6, which are required to classify tumours were available. Similarly, HIF-1α and the PHDs were available in only 188 out of 456 tumours.

### Statistical methods

Correlations were evaluated using either the one-way ANOVA or χ^2^ test where appropriate. Kaplan–Meier survival curves were calculated using tumour recurrence (defined as the first re-appearance of tumour at any site after definitive treatment) and cancer-related death as the endpoints and compared using a log-rank test. Binary logistic regression was used for multivariate analyses and the Cox proportional hazard regression model was used to identify independent prognostic factors for disease-free and overall survival. The analyses were carried out with SPSS 16.0 (SPSS Inc., 233 South Wacker Drive, IL, USA). A two-tailed *P*-value test was used in all analyses and a *P*-value of <0.05 was considered statistically significant.

## Results

### Expression of ER, HER2, CK5/6 and EGFR in invasive breast cancer

Out of 456 tumours 310 (68%) were ER positive; of these, 289 tumours were also HER2 negative and were therefore considered to be of the luminal group (Table 1). Seventy-five tumours (16%) were HER2 positive, regardless of their ER, CK5/6 and EGFR status, and were considered to be of the HER2 group. Of the 169 ER-negative tumours, 62 (14%) were also HER2 negative but CK5/6 and/or EGFR positive; these were considered to be of the basal-like group. Thirty tumours were negative for ER, HER2, CK5/6 and EGFR and were considered to be of the negative group (Table 1) (Supplementary material Figure 1).

### Expression of HIF-1*α* and its regulatory enzymes PHD1, PHD2, PHD3 and FIH

Ninety-four out of 188 tumours (50%) stained positive for HIF-1*α* (Table 1) (Supplementary material Figure 1). Of these, 64 tumours (68%) were of the luminal group, 13 tumours (14%) were of the basal group, 12 (13%) were of the HER2 group and 5 (5%) were of the negative group. Although only 8, 10 and 12% of luminal, basal-like and HER2 tumours, respectively, expressed all three PHD enzymes, the majority expressed at least one PHD enzyme (63, 52 and 72, respectively) (Table 1). None of the six negative tumours expressed all three PHD enzymes although all expressed at least one PHD enzyme. More than half of all the tumours expressed FIH-1 (63% of luminal tumours, 52% of basal tumours, 68% of HER2 tumours and 50% of negative tumours) (Table 1).

### Correlation of CAIX expression in invasive breast carcinoma with clinicopathological parameters

CAIX was positive in 59 out of 407 tumours (14%) (Table 2). The majority of these tumours (26 out of 59 (44%)) were of the basal-like group. Carbonic anhydrase IX expression was significantly correlated with a larger tumour size (*P*=0.005), high tumour grade (*P*<0.001) and ER negativity (*P*<0.001) (Table 2). There was no correlation with patient age, nodal status or HER2 status (*P*>0.05) (Table 2). CAIX expression showed no significant correlation with HIF-1*α* expression (Table 2).

### Correlation of breast tumours stratified by intrinsic subtype with CAIX, HIF-1*α* and its regulatory enzymes

Basal-like tumours were 8.9 times more likely to express CAIX (*P*<0.001, 95% CI 3.86–20.29) than luminal tumours (Table 3). This association is significant (<0.001) even after controlling for the effect of tumour size and grade. There was no significant correlation between breast tumour subtypes and HIF-1*α* or its regulatory enzymes (*P*>0.05).

### Correlation of breast tumours stratified by intrinsic subtype with clinicopathological parameters and survival outcome

Basal-like tumours were associated with high tumour grade (*P*<0.001), but were not correlated with patient age, tumour size or nodal status (*P*>0.05). Basal-like tumours and HER2 tumours shared a similar poor disease-free survival outcome (Supplementary Figure 2) and were associated with a significantly shorter disease-free survival compared with luminal tumours (*P*<0.001, OR 2.58, 95% CI 1.82–3.65) (Negative tumours were excluded from the analysis because of the small sample size but are included the figure).

### CAIX expression and survival in breast cancer patients

**Figure 1:**
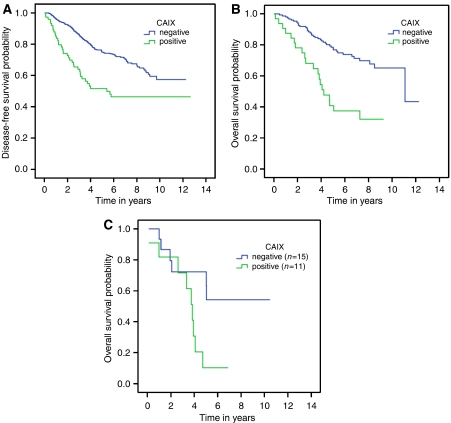
(**A**) Kaplan–Meier disease-free survival curves stratifying patients by CAIX expression (*P*<0.001) (*n*=407). (**B**) Kaplan–Meier overall survival curves stratifying all tumours treated with chemotherapy by expression of CAIX (*P*<0.001) (*n*=427). (**C**) Kaplan–Meier overall survival curves stratifying patients with basal-like tumours treated with chemotherapy by expression of CAIX (*P*=0.03) (*n*=26).

Patients with tumours expressing CAIX had a significantly worse disease-free survival (*P*<0.001) (Figure 1A). There was a significant difference in survival in patients with any type of breast carcinoma treated with chemotherapy stratified by CAIX (*P*<0.001) (Figure 1B). Patients with CAIX-positive basal-like breast cancers who were treated with chemotherapy had a significantly shorter overall survival (*P*=0.03) than treated patients with CAIX-negative basal-like breast cancers (Figure 1C). In the absence of chemotherapy treatment, there was no significant difference in overall survival among the intrinsic subtypes (*P*=0.07), or when basal-like carcinomas were stratified according to CAIX expression (*P*=0.27). There was no significant difference in overall survival in patients not treated with chemotherapy with any type of breast carcinoma (*P*=0.07) or basal-like carcinoma stratified by CAIX (*P*=0.27). In a multivariate Cox regression model patients who had tumours that were CAIX positive and who were treated with chemotherapy had a significantly shorter overall survival (*P*<0.001), whereas CAIX had no effect on survival in patients not treated with chemotherapy (*P*=0.48) (Table 4A and B). To test for treatment interactions, CAIX, chemotherapy and their interaction variables were entered into the Cox regression model as a second block. The interaction of chemotherapy with CAIX was significant in the final model (*P*=0.04) (Table 4C).

## Discussion

mRNA expression profile studies have confirmed the long-standing histological observation that breast cancer is a heterogeneous disease (Perou *et al*, 2000; Sorlie *et al*, 2001, 2003). These studies have broadly classified breast cancers into four categories: luminal A (strongly ER positive), luminal B (also ER positive but proliferative), HER2 and basal-like groups. Although these categories were identified using unsupervised clustering, the groupings also conferred prognostic information, with carcinomas of the basal-like group having a shorter survival than tumours of the luminal group (Sorlie *et al*, 2001) and being resistant to anthracycline-based chemotherapy (Banerjee *et al*, 2006). Expression of the hypoxia-inducible factor-1*α* is also associated with a poor prognosis and resistance to chemotherapy (Teicher *et al*, 1981; Schindl *et al*, 2002; Bos *et al*, 2003), and in view of the association between basal-like breast cancer, necrosis, their aggressive behaviour and poor response to chemotherapy we hypothesised that these tumours may have an intrinsically enhanced hypoxic response. We therefore examined the hypoxic pathway in a series of breast cancers.

Although there is no generally recognised definition of the categories for stratification into intrinsic subgroups (different studies using various markers such as CK5, CK14, CK17, EGFR, KIT, p63 and smooth muscle actin (Nagle *et al*, 1986; Wetzels *et al*, 1991; Malzahn *et al*, 1998; Tsuda *et al*, 1999; van de Rijn *et al*, 2002; Abd El-Rehim *et al*, 2004; Matos *et al*, 2005; Fulford *et al*, 2006) to identify basal-like tumours), in this study we have used the classification developed by Nielsen *et al* (2004). This uses a combination of ER, HER2, CK5 and EGFR to generate four groups, which in this study gives a frequency of basal-like cancers in this cohort similar to that described earlier (van de Rijn *et al*, 2002; Abd El-Rehim *et al*, 2004; Nielsen *et al*, 2004; Matos *et al*, 2005).

Central to the hypoxic response is the transcription factor HIF. We have examined the role of the key pathway members regulating HIF-mediated transcription, including HIF-1*α*, the PHDs, FIH and CAIX in a series of breast cancers classified into intrinsic subtypes as described above (Wykoff *et al*, 2000). We observed that basal-like tumours were many times more likely to express CAIX. Carbonic anhydrase IX is a transmembrane protein involved in maintaining a low pericellular pH through its reversible conversion of carbon dioxide and water into carbonic acid (Pastorek *et al*, 1994; Opavsky *et al*, 1996), and has been shown to maintain survival of breast tumour cells under hypoxic conditions (Potter and Harris, 2003). Carbonic anhydrase IX is not present in normal breast tissues (Bartosova *et al*, 2002), but is upregulated in invasive tumours with increasing intensity, with increasing distance from tumour vessels (Span *et al*, 2003) tightly under the regulation of HIF-1*α* (Grabmaier *et al*, 2004). Carbonic anhydrase IX expression has been shown to correlate with the distribution of pimonidazole, a chemical marker of hypoxia (Opavsky *et al*, 1996; Wykoff *et al*, 2000) and with hypoxia measured by Eppendorf microelectrode in advanced cervical cancer (Loncaster *et al*, 2001). On the basis of these data it has been suggested that CAIX immunohistochemistry is a surrogate marker for detecting hypoxia in tumour samples. Thus, the association between basal phenotype and CAIX supports the notion that these tumours have an enhanced hypoxic response (Wykoff *et al*, 2000; Lal *et al*, 2001).

Nevertheless, despite a strong correlation with CAIX, we did not observe a correlation between basal tumours and HIF-1*α* or between CAIX and HIF-1*α* in this study. This is unlikely to be due to spatial reasons because TMA are ideal for investigating relationships between biomarkers as the same area of tumour is being examined, but is probably due to the difference in half-lives of HIF-1*α* and CAIX. Hypoxia-inducible factor-1*α* is rapidly degraded within minutes of reoxygenation (Jiang *et al*, 1996), whereas CAIX has a half-life of 2–3 days (Turner *et al*, 2002; Rafajova *et al*, 2004). This absence of an association is in agreement with reports (Tomes *et al*, 2003; Sobhanifar *et al*, 2005) of the presence of CAIX without HIF-1*α* expression in perinecrotic regions in solid tumours (Sobhanifar *et al*, 2005).

CAIX expression, like a basal-like phenotype, is associated with an increased risk of recurrence and poorer overall survival (Chia *et al*, 2001). In this series, those patients with tumours of either a basal-like phenotype or expressing CAIX were associated with an adverse outcome. Nevertheless, in this study and in others reported earlier, CAIX expression is also positively correlated with high tumour grade and size (Chia *et al*, 2001; Span *et al*, 2003), the former feature also characteristic of basal tumours. Thus, hypoxic stress may not be part of the basal intrinsic subtype but might be due to an association with grade. Although this cannot be completely discounted, our data show that both basal-like and HER2 cancers are significantly associated with high tumour grade (as expected) but the basal group was more likely to be CAIX positive, whereas the HER2 subtype was less likely to be CAIX positive. Furthermore, in a multivariate analysis including tumour size and grade, basal-like phenotype was still associated with CAIX expression. Thus, the data are strongly suggestive of an enhanced hypoxic drive in this tumour type. This is further supported by interrogation of expression data sets (Oncomine, www.oncomine.org), where there is significant upregulation of not only HIF-1*α* and CAIX but also other HIF-1*α*-regulated genes such as vascular endothelial growth factor-A, BNIP3 and Glut-1. Moreover, patients with basal-like tumours who have not received chemotherapy have a similar prognosis irrespective of CAIX expression, whereas patients with basal-like tumours that are CAIX positive and who have received chemotherapy have a significantly shorter overall survival than patients with basal-like tumours that are CAIX negative. This suggests that CAIX-positive basal-like tumours are particularly resistant to chemotherapy, in contrast to CAIX-negative basal-like tumours, which are able to respond to chemotherapy. A potential mechanism for this action is through trapping of chemotherapeutic agents outside the cell when the extracellular environment is acidic (Mahoney *et al*, 2003).

The resistance effect of CAIX/hypoxia seems to hold irrespective of tumour subtype as, unlike patients not treated with chemotherapy, treated patients with any tumour phenotype positive for CAIX also had a significantly worse survival than patients with tumours negative for CAIX in the multivariate analyses. This is in keeping with our studies in the neoadjuvant context, in which CAIX was associated with a shorter relapse-free survival in patients treated with CMF (Generali *et al*, 2006a, 2006b), and in patients treated in the adjuvant setting, in which CAIX measured by RT–PCR was associated with chemoresistance (Span *et al*, 2003). Thus, these data suggest that CAIX is predictive rather than prognostic.

Molecular genetic studies of basal-like breast carcinomas have shown that tumours with a basal-like phenotype have a higher number of unbalanced chromosomal changes and rate of loss of heterozygosity than luminal-type breast tumours (Korsching *et al*, 2002; Jones *et al*, 2004). In addition, basal-like tumours are a distinctive feature of BRCA1-associated breast cancers. Although BRCA1 is involved in a large number of cellular processes, it is the maintenance of genomic stability that has been proposed to be the principal factor underlying cancer predisposition in mutation carriers (Baldeyron *et al*, 2002). Thus, it is also of interest that hypoxia is recognised to induce genomic instability (Cheng and Loeb, 1993; Sutherland, 1998; Semenza, 2000; Chan *et al*, 2008), and it is interesting to speculate whether the genomic alterations observed in basal-like breast carcinomas may partly be the result of the enhanced hypoxic response in this subtype. Nevertheless, these genomic studies have been carried out on a small number of tumours (Jones *et al*, 2001, 2004) and additional data are required to support this thesis.

In summary, the more aggressive phenotype seen in basal-like tumours may be at least partly due to the intrinsically enhanced hypoxic properties of basal-like breast cancers. It suggests that as this tumour type is particularly difficult to treat, because they are both hormonal non-responsive and resistant to chemotherapy, targeting HIF with small molecule inhibitors, HRE-regulated gene therapies, carbonic anhydrase inhibitors or the use of bio-reductive drugs may be of particular use in treating this aggressive cancer (Supuran, 2008).

## Figures and Tables

**Table 1:** Correlation analysis of 456 invasive breast carcinomas with clinicopathological parameters and CA9

	**Luminal (*n*=289)**	**HER2 (*n*=75)**	**Basal (*n*=62)**	**Negative (*n*=30)**	***P*-value**
*Patient age*					0.64
Median (years)	56.0	55.2	54.0	52.7	
					
*Tumour size*					0.40
Median (mm)	18.0	22.0	22.5	20.5	
					
*Tumour grade*					<0.001
1	71	4	2	4	
2	156	16	12	12	
3	58	54	47	14	
					
*Nodal status*					0.75
Negative	162	40	34	17	
Positive	125	34	28	13	
					
*CAIX*					<0.001
Negative	242	27	57	22	
Positive	14	26	16	3	
					
*Nuclear HIF1α*					0.53
Negative	54	10	13	1	
Positive	64	12	13	5	
					
*Cytoplasmic PHD1*					0.76
Negative	68	10	15	3	
Positive	51	12	11	3	
					
*Cytoplasmic PHD2*					0.41
Negative	71	9	16	4	
Positive	47	11	7	2	
					
*Cytoplasmic PHD3*					0.87
Negative	70	13	14	3	
Positive	43	10	11	3	
					
*Nuclear FIH-1*					0.51
Negative	45	6	12	3	
Positive	80	17	15	3	

CAIX=carbonic anhydrase IX; PHD=prolyl hydroxylase.

*n*<456 because data are not available.

**Table 2:** Correlation analysis of CAIX expression and clinicopathological parameters

	**CAIX positive (*n*=59)**	**CAIX negative (*n*=348)**	***P*-value**
*Patient age*			0.96
Median (years)	55.0	57.0	
			
*Tumour size*			0.005
Median (mm)	25.0	19.0	
			
*Tumour grade*			< 0.001
1	0	96	
2	19	189	
3	56	154	
			
*Nodal status*			0.168
Negative	34	242	
Positive	40	195	
			
*ER status*			<0.001
Negative	54	110	
Positive	19	326	
			
*HER2 status*			0.065
Negative	43	291	
Positive	16	57	
			
*Nuclear HIF-1α*			0.13
Negative	6	54	
Positive	15	62	

CAIX=carbonic anhydrase IX; PHD=prolyl hydroxylase; HIF=hypoxia-inducible factor.

*n*<456 because data are not available.

**Table 3:** Multivariate analysis binary logistic regression model of the effect of CAIX expression on tumour subtype, using luminal tumours as a baseline (*n*=406)

	**Odds ratio**	**95% CI**	***P*-value**
Tumour size	1.00	0.98–1.03	0.79
Grade	2.7	1.45–5.00	0.002
Basal tumours	8.9	3.86–20.29	<0.001
HER2 tumours	2.7	1.16–6.21	0.02
Negative tumours	1.6	0.40–6.09	0.52

CAIX=carbonic anhydrase IX.

**Table 4A:** Cox regression model, overall survival, all breast cancers treated with chemotherapy (*n*=182)

	**Odds ratio**	**95% CI**	***P*-value**
Nodal status	1.77	0.62–5.05	0.29
Grade	2.38	1.36–4.164	0.002
CAIX	3.20	1.79–5.701	<0.001
Size	1.04	1.02–1.058	<0.001

CAIX=carbonic anhydrase IX

**Table 4B:** Cox regression model, overall survival, all breast cancers treated with chemotherapy (*n*=262)

	**Odds ratio**	**95% CI**	***P*-value**
Nodal status	2.27	1.04–4.96	0.04
Grade	1.90	1.12–3.25	0.02
CAIX	1.33	0.60–2.95	0.48
Size	1.04	1.02–1.06	<0.001

CAIX=carbonic anhydrase IX

**Table 4C:** Cox regression model, overall survival, all breast cancers with CAIX and chemotherapy as interaction variables (*n*=441)

	**Odds ratio**	**95% CI**	***P*-value**
Grade	1.98	1.34–2.92	0.001
Age	1.00	0.99–1.02	0.65
Size	1.04	1.03–1.05	<0.001
Oestrogen receptor	0.74	0.46–1.20	0.22
Nodal status	2.22	1.15–4.26	0.02
			
*Additions to model*			
CAIX	1.09	0.49–2.42	0.83
Chemotherapy	0.75	0.38–1.49	0.41
CAIX × chemotherapy	2.65	1.03–6.82	0.04

CAIX=carbonic anhydrase IX.
