# Supplementary material for: The key hypoxia regulated gene CAIX is upregulated in basal-like breast tumours and is associated with resistance to chemotherapy
Source: Br J Cancer. 2009 Jan 22;100(2):405–11. doi: 10.1038/sj.bjc.6604844 (PMC2634728; doi:10.1038/sj.bjc.6604844)

**Supplemental materials**

Table 1. Antibodies used in study.

| **Antigen** | **Antibody** | **Dilution** | **Antigen retrieval** | **Incubation time (min)** |
| --- | --- | --- | --- | --- |
| CK 5/6 | DakoCytomation, A/S, DK | 1:100 | Pressure cook 1.5min in 0.1mM citrate pH 6.0 | 30 |
| HIF-1α | ESEE122(Tal*ks et* al, 2000) | 1:100 | Pressure cook 3min in EDTA pH8.0 |  |
| PHD1 | PHD112 (Appelho*ff et* al, 2004) | Neat  Supernatant | None | 90 |
| PHD2 | 76a (Appelho*ff et* al, 2004) | Neat  Supernatant | None | 90 |
| PHD3 | EG188e (Appelho*ff et* al, 2004) | Neat  Supernatant | None | 90 |
| FIH-1 | FIH162c (Stol*ze et* al, 2004) | Neat  Supernatant | None | 90 |
| CAIX | M75 (Pastoreko*va et* al, 1997) | 1:50 | None | 60 |

Appelhoff RJ, Tian YM, Raval RR, Turley H, Harris AL, Pugh CW, Ratcliffe PJ, Gleadle JM (2004) Differential function of the prolyl hydroxylases PHD1, PHD2, and PHD3 in the regulation of hypoxia-inducible factor. *J Biol Chem* **279:** 38458-65

Pastorekova S, Parkkila S, Parkkila AK, Opavsky R, Zelnik V, Saarnio J, Pastorek J (1997) Carbonic anhydrase IX, MN/CA IX: analysis of stomach complementary DNA sequence and expression in human and rat alimentary tracts. *Gastroenterology* **112:** 398-408

Stolze IP, Tian YM, Appelhoff RJ, Turley H, Wykoff CC, Gleadle JM, Ratcliffe PJ (2004) Genetic analysis of the role of the asparaginyl hydroxylase factor inhibiting hypoxia-inducible factor (HIF) in regulating HIF transcriptional target genes. *J Biol Chem* **279:** 42719-25

Talks KL, Turley H, Gatter KC, Maxwell PH, Pugh CW, Ratcliffe PJ, Harris AL (2000) The expression and distribution of the hypoxia-inducible factors HIF-1alpha and HIF-2alpha in normal human tissues, cancers, and tumor-associated macrophages. *Am J Pathol* **157:** 411-21

**Figure 1**. **Immunohistochemisty of CK5/6, HIF-1α, CAIX and HER2 in invasive breast carcinoma.** A. Positive CK5/6 staining in a basal-like tumor. B. Positive nuclear HIF-1α staining in a basal-like tumor. C. Strong membranous CAIX staining in a basal-like tumor. D. Negative CAIX staining in a luminal tumor. E. Negative CK5/6 staining in a luminal tumor. F. Strong complete membranous HER2 staining in a HER2 tumor.


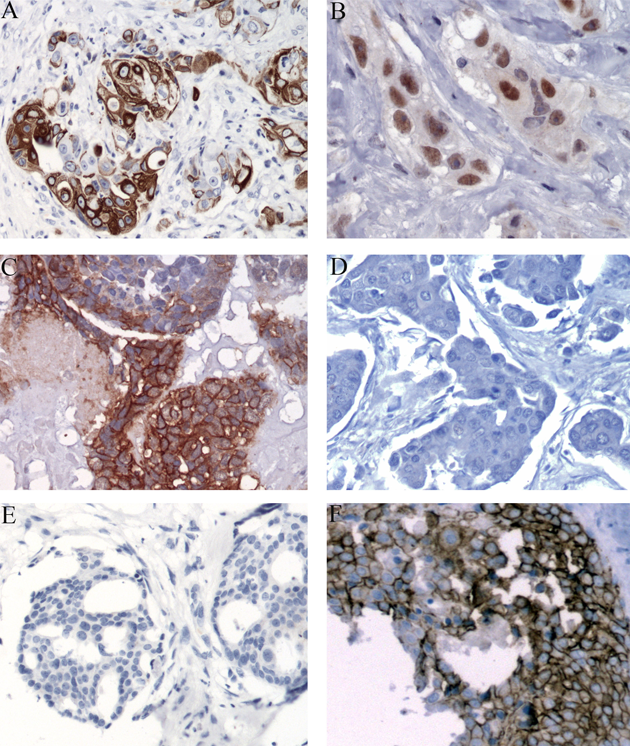


**Figure 2**. Kaplan Meier disease-free survival curves stratifying patients by tumor subgroups (n=456).


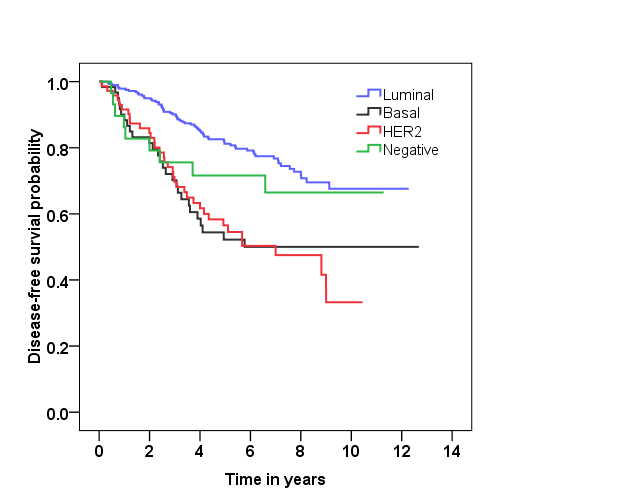

Supplement: Supplementary Material [file 6604844x1.doc]
